# Ginsenoside Rd protects transgenic *Caenorhabditis elegans* from β-amyloid toxicity by activating oxidative resistant

**DOI:** 10.3389/fphar.2022.1074397

**Published:** 2022-12-15

**Authors:** Lihan Mi, Meiling Fan, Tianjia Liu, Donglu Wu, Yang Wang, Fuqiang Li, Yong Cai, Zhidong Qiu, Da Liu, Lingling Cao

**Affiliations:** ^1^ School of Pharmacy, Changchun University of Chinese Medicine, Changchun, China; ^2^ Affiliated Hospital of Changchun University of Chinese Medicine, Changchun, China; ^3^ Scientific Research Department, The Third Affiliated Hospital of Changchun University of Chinese Medicine, Changchun, China; ^4^ Key Laboratory of Effective Components of Traditional Chinese Medicine, Changchun, China; ^5^ School of Clinical Medical, Changchun University of Chinese Medicine, Changchun, China; ^6^ School of Life Sciences, Jilin University, Changchun, China

**Keywords:** ginsenoside rd, alzheimer’s disease, oxidative resistant, β-amyloid, *C. elegans*

## Abstract

Alzheimer’s disease (AD) is a serious public health issue but few drugs are currently available for the disease, and these only target the symptoms. It is well established that oxidative stress plays a crucial role in AD, and there is compelling evidence linking oxidative stress to β-amyloid (Aβ). An exciting source of potential new AD therapeutic medication possibilities is medicinal plants. Ginsenoside Rd (GS-Rd) is one of the main bioactive substances in ginseng extracts. In our study, we used a network pharmacology analysis to identify overlapping GS-Rd (therapeutic) and AD (disease)-relevant protein targets, gene ontology (GO) and bio-process annotation, and the KEGG pathway analysis data predicted that GS-Rd impacts multiple targets and pathways, such as the MAPK signal pathway and the JAT-STAT3 signaling pathway. We then assessed the role of GS-Rd in *C. elegans* and found that GS-Rd prolongs lifespan, improves resistance to heat stress, delays physical paralysis and increases oxidative stress responses. Overall, these results suggest that GS-Rd protects against the toxicity of Aβ. The RNA-seq analysis revealed that GS-Rd achieves its effects by regulating gene expressions like *daf-16* and *skn-1*, as well as by participating in many AD-related pathways like the MAPK signaling pathway. In addition, in CL4176 worms, GS-Rd decreased reactive oxygen species (ROS) levels and increased SOD activity. Additional research with transgenic worms showed that GS-Rd aided in the movement of DAF-16 from the cytoplasm to the nucleus. Taken together, the results indicate that GS-Rd significantly reduces Aβ aggregation by targeting the MAPK signal pathway, induces nuclear translocation of DAF-16 to activate downstream signaling pathways and increases resistance to oxidative stress in *C. elegans* to protect against Aβ-induced toxicity.

## 1 Introduction

Alzheimer’s disease (AD) is a common clinical neurodegenerative disease characterized by progressive cognitive impairment and memory loss (Tatulian, 2022). The most widely studied core mechanism is the proteolytic fission of amyloid-β precursor protein (APP). Typically, the cleavage of APP occurs by the sequential action of α-secretase and γ-secretase to produce non-amyloidogenic products (the non-amyloidogenic pathway) (Ashton et al., 2018). AD is pathologically characterized by senile plaques formed by extracellular Amyloid-β (Aβ) (Pinheiro and Faustino, 2019) peptide and Intracellular Neurofibrillary Tangles (NFT) (Roda et al., 2022) formed by hyperphosphorylated tau protein. Amyloid cerebrovascular disease and hemorrhagic stroke are also caused by excessive accumulation of Aβ in many AD cases (Jastrzebski et al., 2015). Aβ protein remains the most important target in current AD research.

**FIGURE 1:**
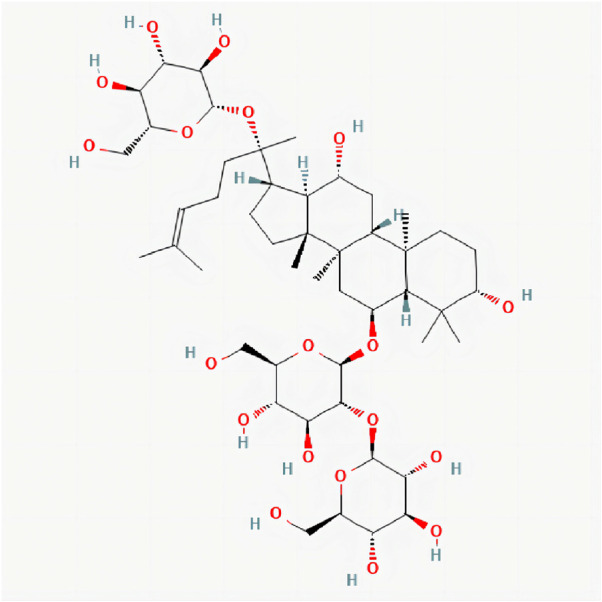
2D chemical structure of Ginsenoside Rd. Structure of Ginsenoside Rd cited from pubchem (pubchem CID, 24721561). The molecular formula is C_48_H_82_O_19_.

Ginsenoside is one of the main active components of the Araliaceae plant ginseng (Panax ginseng C. A. Mey.). GS-Rd (ginsenoside Rd) is one of its monomer components, the chemical structure of GS-Rd is shown in Figure 1. In recent years, GS-Rd has received extensive attention due to its good pharmacological activity. Researchers found that GS-Rd can activate the mRNA and protein expression of the HIF-1ɑ signaling pathway and the synaptic plasticity-related regulators, having a significant therapeutic effect on depression (Li et al., 2022). GS-Rd improves vascular damage caused by diabetes by activating AMPK and promoting the expression of SIRT1 and has a good protective effect on blood vessels (Tang et al., 2022); GS-Rd performs a protective role in EAN (Experimental autoimmune neuritis) nerve injury by modulating monocyte conversion and might be a novel preventive intervention for GBS (Guillain-Barre Syndrome) (Ren et al., 2021). In recent years, the neuroprotective effect of ginsenoside Rd has been confirmed, and it has received more and more attention. Ginsenoside Rd prevented TMT-induced cell apoptosis *via* the regulation of B cell lymphoma 2 (Bcl-2), BCL-2-like protein four and caspase-3 (Hou et al., 2017). The neuroprotective function of GS-Rd in acute ischemic stroke may be partly through the up-regulation of NEIL1 and NEIL3 expressions (Yang et al., 2016). Further studies have shown that GS-Rd has a certain role in the treatment of AD and that GS-Rd has a significant neuroprotective effect on rats insulted by Aβ1-40 (Liu et al., 2012). Furthermore, GS-Rd can be used as alternative drug therapy for AD patients in the prevention and treatment of their memory dysfunction (Liu et al., 2015), Ling Li et al. (Li et al., 2021) demonstrate that GS-Rd is capable of inhibiting the Aβ-induced tau phosphorylation by altering the functional balance of GSK-3β and CDK5/P25 in the olfactory bulb, spinal cord and telencephalon. However, the specific therapeutic molecular mechanism of GS-Rd in AD remains largely unknown. It is clear that GS-Rd needs to continue to be explored in the prevention and treatment of AD and has important research value.

In the past 10 years, the emerging discipline of network pharmacology has been widely used in the discovery of drugs and active compounds in traditional Chinese medicine. Network pharmacology has also provided new scientific and technological support for the research and development of new drugs (Berger and Iyengar, 2009). To explore hypotheses about the therapeutic effects of GS-Rd on AD, our study used a network pharmacology analysis. The protein targets, biological processes and biological pathways of GS-Rd in the treatment of AD were predicted. Biological network construction and visualization analysis were used to identify complex interactions between GS-Rd and AD-related targets. The binding of GS-Rd to potential target proteins was predicted in combination with molecular docking methods.

Based on these analyses, we utilized the *Caenorhabditis elegans* (*C.elegans*) AD model to further clarify the pharmacological effects and mechanisms in the GS-Rd treatment of AD. Due to its short lifespan, 42% of its genes being related to human genes, and its possessing an intact nervous system, *C. elegans* is frequently used as a model for fundamental biology research (Wang et al., 2014). The transgenic *C. elegans* strain CL4176 expresses human Aβ1-42 in muscle cells under a temperature-inducible system: above 25°C they become rapidly paralyzed (Alvarez et al., 2022). This serves as a model for studying Aβ neurotoxicity in AD. Zhang XG (Zhang et al., 2016) demonstrated that GS-Rd delayed Aβ-induced paralysis in the transgenic CL4176 strain. In this study, CL2006 worms were used for heat resistance tests and the detection of anti-Aβ effects of GS-Rd; Paralysis experiments, Aβ mRNA level detection and RNA-seq studies were carried out to verify the mechanism of GS-Rd in treating AD; The CL4176 strain was also used to measure the levels of ROS and SOD to investigate why GS-Rd delayed worm paralysis. TJ356 worms were used in our study to investigate the impact of GS-Rd on DAF-16 distribution since they carry the DAF-16:GFP fusion protein (Wang et al., 2022). To our knowledge, this is the first time that network pharmacology has been used to evaluate the potential therapeutic mechanism of GS-Rd in the treatment of AD (in conjunction with *in vivo* validation in *C. elegans* models).

## 2 Materials and methods

### 2.1 Reagents and strains

Ginsenoside Rd (with a purity >98%) was purchased from Chengdu Must Biotechnology, A stock solution of GS-Rd (50 mM) was prepared with DMSO (Sigma, D2650). The following strains were used in this work: CL4176 (dvIs27 [myo-3p:Aβ (1–42):let-851 30 UTR) + rol-6 (su1006)]); CL 2006 (dvIs2 [pCL12 (unc-54/human Aβ peptide 1–42 minigene) + pRF4]) and TJ356 (zls356 [daf-16p:daf-16a/b:GFPC + rol-6). These were provided by the *Caenorhabditis* Genetic Center (CGC). All worms were propagated at 16 °C except TJ356, which was propagated at 20 °C on nematode growth medium (NGM) and seeded with the standard food resource of *E. coli* OP50.

### 2.2 Prediction of potential GS-Rd-related targets

Potential GS-Rd-related targets were obtained from the Swiss Target Prediction database (http://www.swisstargetprediction.ch/) and the PharmMapper Server (http://www.lilab-ecust.cn/pharmmapper/). In the Swiss Target Prediction database, potential GS-Rd-related targets were retrieved by the structure of GS-Rd in Figure 1 and species were limited to “*Homo sapiens*”. All targets obtained in both the Swiss Target Prediction database and PharmMapper were selected as potential GS-Rd-related targets.

### 2.3 Potential targets of GS-Rd against AD

AD-related targets were identified in the Human Gene Database (GeneCards, https://www.genecards.org/), the Online Mendelian Inheritance in Man database (OMIM, https://www.omim.org/), and the Therapeutic Target Database (TTD, http://db.idrblab.net/ttd/) using keywords “Alzheimer’s disease”. The Uniprot (https://www.uniprot.org/) database was used to unify target names and exclude non-human targets in preparation for the topological analysis of the subsequent protein interaction (PPI) network. Subsequently, Venny 2.1.0 (https://bioinfogp.cnb.csic.es/tools/venny/) was applied to screen the common targets of GS-Rd and AD which were defined as the potential targets of GS-Rd against AD in the present research.

### 2.4 Constructing component-target networks

The targets obtained in 2.2 are used in Cytoscape 3.9.1 to create a comprehensive “component-target” network to visualize the relationship between drug component targets and disease targets, enabling in-depth analysis of the potential therapeutic mechanisms of GS-Rd in AD.

### 2.5 PPI network construction, analysis, and core gene screening

The protein-protein interaction (PPI) network of potential targets of GS-Rd against AD was constructed by using the STRING database (https://cn.string-db.org/), using common targets of GS-Rd and AD with the minimum required interaction score ≥0.900, and with the species limited to “*Homo sapiens*”, the network edge was based on “evidence”. Proteins independent of (not connected to) the network were excluded, and a network topology analysis was applied to identify proteins with high degrees of connectedness. The CSV file of this network was imported into Cytoscape 3.9.1 to filter out the 10 core targets. Using Cytoscape’s plug-in cytohubba to calculate the ranking according to the score by the MCC method, The 10 core targets were then subjected to KEGG enrichment analysis.

### 2.6 GO and KEGG pathway enrichment analysis

**FIGURE 2:**
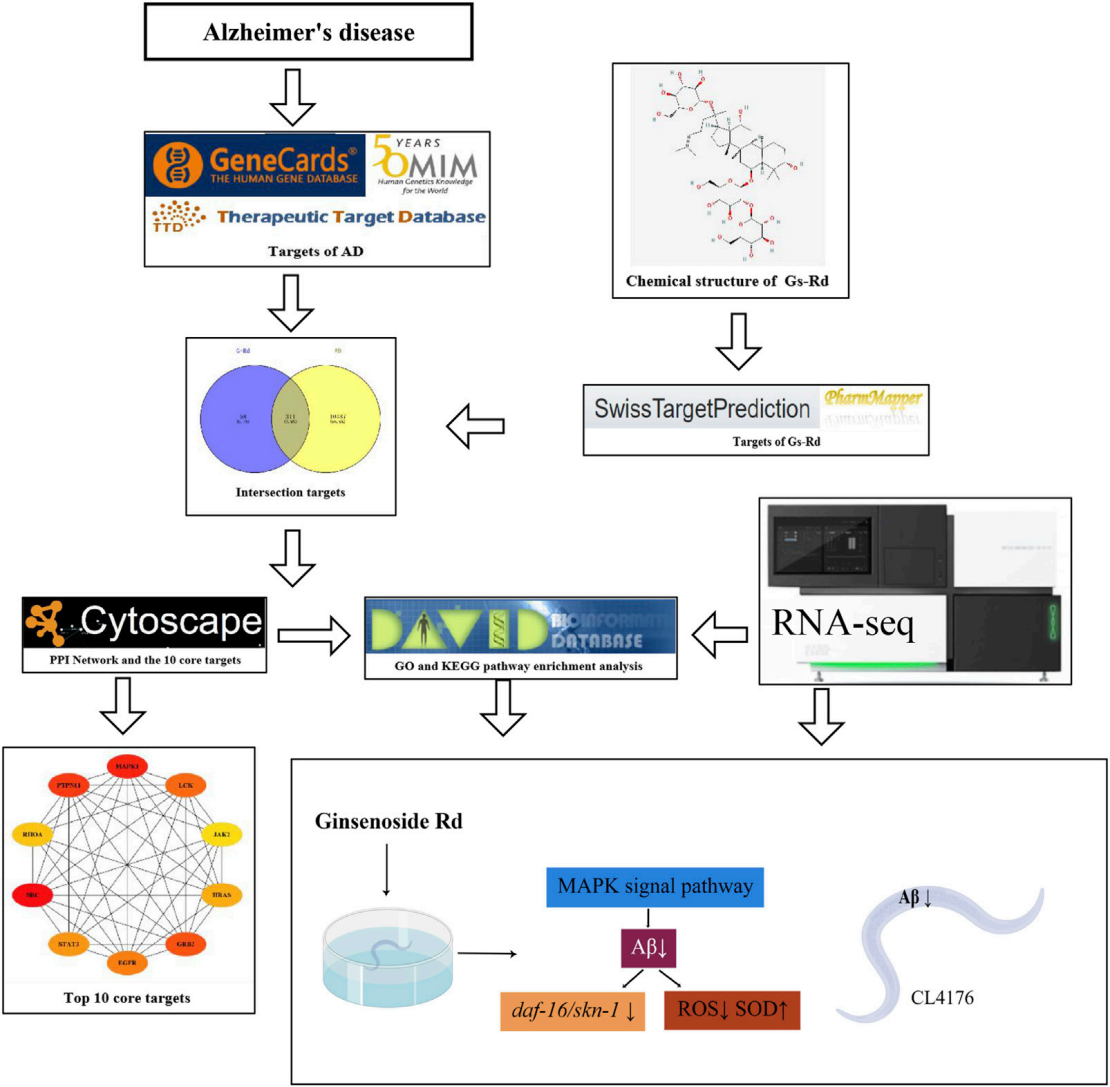
The entire structure of this study is based on the methods of network pharmacology and experimental verification using *C. elegans.*

The GO and KEGG pathway enrichment of the common targets of GS-Rd and AD were analyzed on the DAVID Bioinformatics Resource (DAVID, https://david.ncifcrf.gov/). The results are visualized by the Omicshare cloud platform online tool, *p* < 0.05 indicates that the difference is statistically significant. Figure 2 displays the whole network pharmacology framework.

### 2.7 Component-target molecular docking

By searching the PubChem website (https://pubchem.ncbi.nlm.nih.gov/), the SDF structure file of the compound was obtained, and Open Babel 2.3.2 software was used to convert SDF files into PDB files that were retrieved from the Protein Data Bank (http://www.rcsb.org/pdb) database to obtain the receptor proteins JAK2(PDBID: 6G3C), STAT3(PDBID: 6NJS), GRB2(PDBID: 1JYQ) and MAPK1 (PDBID: 6SLG), and PYMOL 2.3.4 software was used to perform operations such as removing water and ligands from the receptor protein. Furthermore, AutoDock Vina 1.1.2 was used for the molecular docking of receptor protein and the ligand small molecule. A binding energy of less than 0 indicates that the ligand and the receptor can bind spontaneously. A binding energy of −5.0 kJ/mol was selected as the basis for screening GS-Rd therapeutic targets for AD.

### 2.8 Lifespan assay

Before conducting the experiments, all worms were maintained at a permissive temperature and grown for at least two generations in the presence of food to ensure health. Synchronized L1 worms were fed with OP50 and grown to young adult, and then 30 worms were transferred to a new plate. Lifespan analyses were conducted at 16 °C (CL 2006): worms were tapped every day and scored as dead when they did not respond to the platinum wire pick. All of the lifespan assays were repeated three times. Survival plots, *p* values (log-rank), and proportional hazards were determined by using GraphPad Prism seven software.

### 2.9 Heat resistance assay and heat recovery assay

For heat resistance assays, synchronous strains of CL2006 worms at the L4 stage were transferred to plates with or without GS-Rd and incubated at 35 °C. Dead worms were counted every 1 h. A Heat Recovery Assay was carried out at 35°C for 7 h, then they were transferred to 16 °C and the number of dead worms was measured after 24 and 48 h. For each assay, at least 30 synchronous worms were studied and three independent trials were performed. For statistical analysis, *p* values were calculated by a two-tailed *t-test*, each consisting of control and experimental worms at the same time.

### 2.10 Paralysis assay

Transgenic *C. elegans* of temperature-sensitive Aβ strain CL4176 maintained at 16 °C were egg-synchronized onto the NGM plates containing 0, 5, 20, 80,160 μM GS-Rd. They were all diluted at indicated final concentrations in 0.1% DMSO according to their maximum saturated solubility. The worms were induced to express human Aβ1-42 until they were at L3 stage larvae by up-shifting the incubation temperature from 16 °C to 25 °C for 34 h. The transgenic worms were scored at 2-h intervals for paralysis until all worms in the negative control group were paralyzed. Data were representative of three different experiments with 30 worms each. Each worm was gently touched with a platinum loop to identify the paralysis; worms were considered to be paralyzed if they did not move or only moved their heads (Dostal et al., 2010).

### 2.11 RNA isolation and quantitative PCR

To prepare worm RNA samples, worms were picked up and placed in clean plates to minimize contamination. Then, approximately 200 worms were suspended in 50 μL M9 buffer. The total RNA of the worms was prepared by using an RNAiso Plus (Takata, No.9108), according to the manufacturer’s instructions. The cDNA was generated with oligo (dT) primers by using the RevertAid RT (Thermo Scientific™ No.K1691). The Quantitative Real-Time PCR was carried out using the SYBR Green Real-time PCR Master Mix (TaKaRa, No. RR420A) on a QuantStudloM five Real-Time PCR Instrument. The mRNA expression levels of Aβ were normalized by the expression of *act-1*. We used *act-1* as an internal control for equal RNA loading. The primer sequences for PCR are shown in the Supplementary Data.

### 2.12 RNA-seq and pathway enrichment analysis

The total RNA was extracted from DMSO and GS-Rd groups of CL4176 worms using RNAiso Plus reagent, and each group was prepared with two parallel replicates and 500 worms. Later, all of the samples were sent to BGI Corporation (Shenzhen, China) for further RNA-seq detection and analysis *via* BGISEQ-500 sequencer. The pathway analysis for differentially expressed genes (DEGs) was performed based on the KEGG database. The data were analyzed on the Dr Tom network platform of BGI (http://report.bgi.com).

### 2.13 ROS and SOD assay

The CL4176 worms, which had become L4 larvae, were treated with GS-Rd for 2 days at 16°C. They were then warmed up to 25 °C and incubated for 24 h; about 1,000 worms were collected into 200 μL M9 Buffer in Eppendorf tubes. The worms were sonicated (Xinzhi, JY92-IIN) and pipetted into wells of 96-well plates containing a final concentration of 10 μM/L DCF-DA (Reactive Oxygen Species Assay Kit, Beyotime). Samples were read in a Gemini EM fluorescence microplate reader (Molecular Devices) at 37°C with an excitation of 485 nm and an emission of 530 nm. The remaining worm supernatant solution was used for the SOD assay (Total Superoxide Dismutase (T-SOD) assay kit, Nanjing Jiancheng) according to the instructions. Samples were read in a Gemini EM fluorescence microplate reader with an excitation of 550 nm.

### 2.14 Cellular localization of DAF-16::GFP analysis

The TJ356 worms were incubated for 4h at 20°C and allowed to lay eggs. Their surviving progeny were treated with GS-Rd (0, 5, 20, 80 μM) and grown into young adults, with there being 15 nematodes per NGM plate. The patterns of DAF-16:GFP cellular localization were assessed as “high”, “medium” and “low”. “High” indicates that a strong DAF-16:GFP signal was present in all nuclei; “Medium” indicates that the DAF-16:GFP signal was present nuclear in some tissues, but cytoplasmic in the majority of tissues; “Low” indicates that the DAF-16:GFP signal was present completely cytoplasmic (Wang et al., 2020).

### 2.15 Statistical analysis

Statistical analysis was performed using GraphPad Prism seven software. Values were expressed as means ± standard deviation. A one-way analysis of variance (ANOVA), followed by Dunnett’s *t-test*, was used to analyze the mean differences among groups compared with the control groups. Values of *p* < 0.05 were considered statistically significant. For lifespan assays, *p* values were determined by a log-rank test.

## 3 Results

### 3.1 Potential target gene analysis of GS-Rd against AD

**FIGURE 3:**
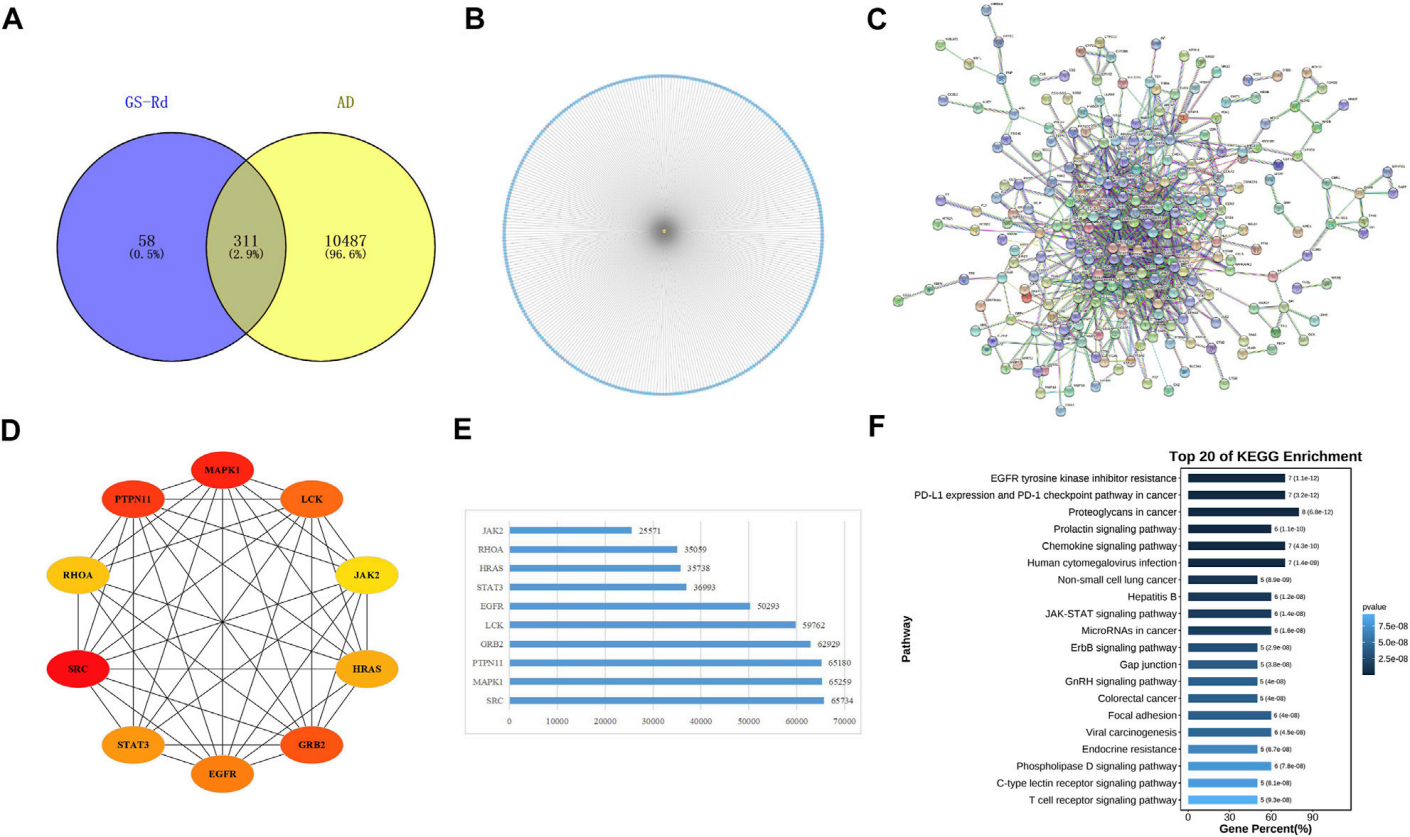
Targets genes analysis of GS-Rd in regulating AD. **(A)** Wayne diagram of GS-Rd and AD candidate targets. **(B)** GS-Rd- targeting-AD network. **(C)** The PPI network of potential targets of GS-Rd against AD. The “node” in the network represents the target protein, and the “edge” represents the interaction between the target proteins. Edges indicate the importance of the target protein corresponding to the node in the network. **(D)**The interaction diagram of the top 10 targets of GS-Rd against AD. **(E)** MCC calculates core target score statistics. **(F)** KEGG enrichment analysis of the core targets.

GS-Rd component targets and AD disease targets were obtained separately through the Swiss Target Prediction database and the PharmMapper Server. A total of 311 cross-targets of GS-Rd and AD were obtained by using Venn diagram tools; the data is shown in Figure 3A. Subsequently, a network map of 311 protein targets was generated using Cytoscape software (Figure 3B), which includes one component node (treatment target) and 311 disease nodes (disease target). This result explains that GS-Rd can treat AD through multi-target regulation.

A PPI network was acquired from STRING to illustrate the relationships between the 310 common targets of GS-Rd and AD (Figure 3C). The PPI network has 310 nodes and 894 edges. The average node degree of the constructed network is 5.77, and the local clustering coefficient is 0.389. This result fully reflects the complexity of the molecular mechanism of GS-Rd treatment of AD; the node pairs with a confidence value higher than 0.98 are listed in Table 1; HSP90AA1, JAK2, STAT3, GRB2, PTPN11, MAPK1 and EGFR exhibited significant interactions with other target proteins.

**TABLE 1:** Nodes from AD targets’ PPI network (Score>0.98).

sNode1	Node2	Coexpression	Experimentally determined interaction	Database annotated	Automated textmining	Combined score
JAK2	STAT3	0.053	0.884	0.9	0.995	0.999
JAK2	PTPN11	0.062	0.8	0.9	0.983	0.999
GRB2	JAK2	0	0.728	0.9	0.982	0.999
EGFR	STAT3	0	0.931	0.9	0.989	0.999
EGFR	PTPN11	0	0.941	0.9	0.986	0.999
EGFR	GRB2	0	0.998	0.9	0.993	0.999
SRC	STAT3	0.061	0.883	0.9	0.986	0.999
MAPK1	STAT3	0.062	0.888	0.9	0.707	0.996
HRAS	SRC	0.105	0.668	0.9	0.89	0.996
GRB2	PTPN11	0.095	0.948	0.9	0.995	0.996
EGFR	HRAS	0.052	0.52	0.9	0.936	0.996
GRB2	HRAS	0.089	0.5	0.9	0.901	0.994
RHOA	SRC	0.084	0.14	0.9	0.929	0.993
HRAS	PTPN11	0.086	0.588	0.9	0.811	0.991
LCK	STAT3	0	0.71	0.9	0.615	0.987
GRB2	MAPK1	0.062	0.213	0.9	0.833	0.986
HRAS	MAPK1	0.111	0.686	0.8	0.768	0.985
PTPN11	SRC	0.064	0.747	0.9	0.99	0.985
GRB2	SRC	0.108	0.788	0.9	0.991	0.984

Next, Cytoscape 3.9.1 was used to obtain 10 core targets (Figure 3D), such as MAPK1, JAK2, EGFR, STAT3 and GRB2 after doing a calculation with the MCC method (Chin et al., 2014). The network’s nodes were then rated according to their network capabilities (Figure 3E); these core genes are more representative of the main biological effect-producing function of GS-Rd. Then, they were subjected to KEGG enrichment analysis and visualized with the help of Omicshare cloud platform online tools (Figure 3F); these core genes are mainly distributed in “proteoglycans in cancer”, “JAK-STAT signaling pathway”, “MicroRNAs in cancer” and “EGFR tyrosine kinase inhibitor resistance”. It suggests that these 10 core targets may play an important role in the pharmacological action of GS-Rd against AD.

### 3.2 GO biological annotation and KEGG pathway enrichment analysis

**FIGURE 4:**
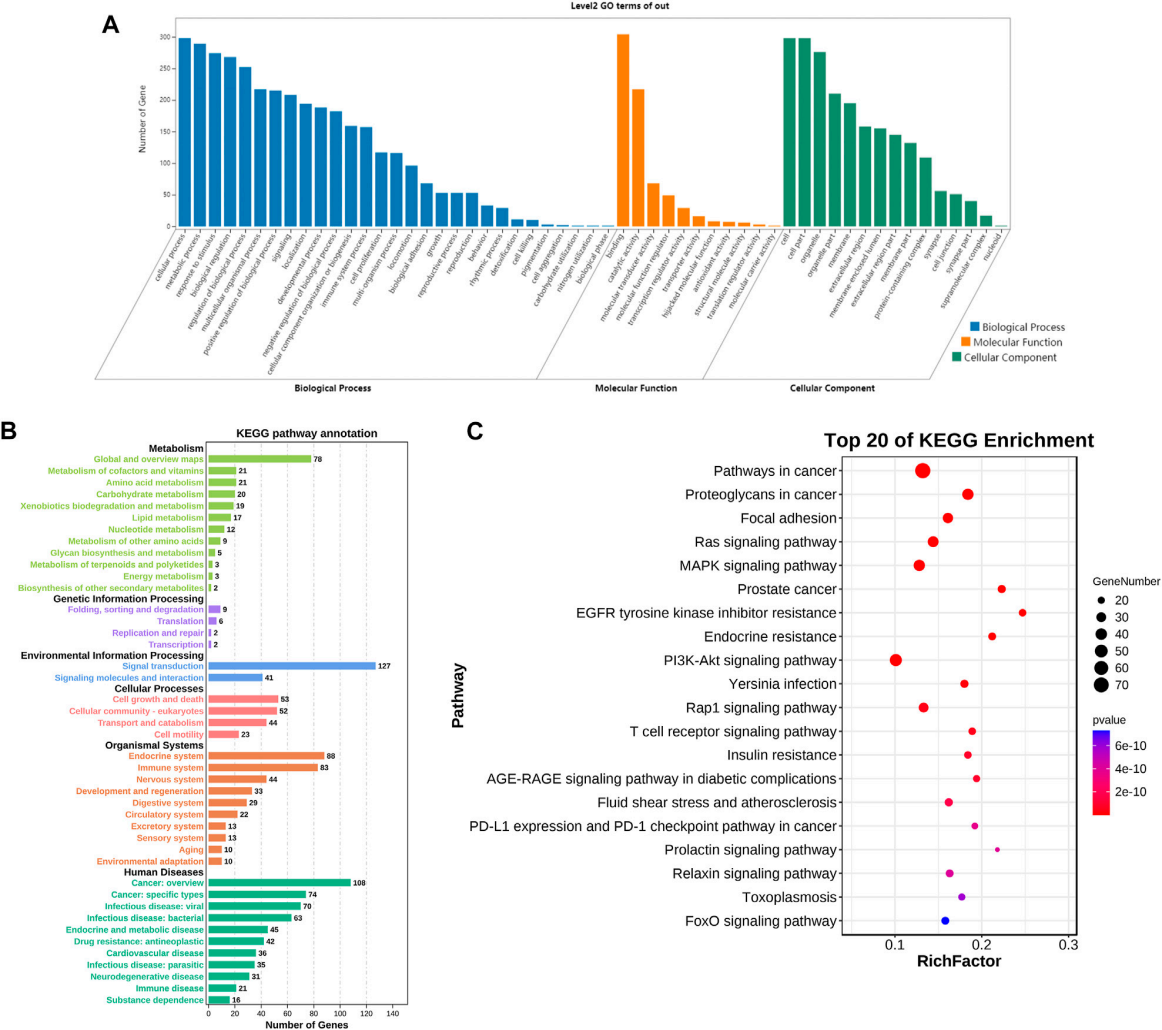
GO bio-annotation and KEGG pathway enrichment analysis of GS-Rd for AD treatment. **(A)** histogram of significance for GO functional enrichment. **(B)** Statistics on KEGG pathway enrichment. **(C)** Top 20 putative targets of KEGG pathway enrichment.

To analyze the biological processes and molecular functions of potential targets involved in AD, the enrichment analysis of GO terms is shown in Figure 4A, including 29 biological processes (BPs), 11 molecular functions (MFs), and 15 cellular components (CCs). The target genes of GS-Rd were predominantly enriched in “cellar process,” “metabolic process,” “response to stimulus,” and “biological regulation,” and the molecular functions involved were molecular binding, catalytic activity, and transcription regulator activity. These findings suggest that GS-Rd intervention in AD may have an impact on metabolic process, stimulus-response and other functions, as well as transcriptional regulation (See supplementary Table 2).

Meanwhile, the KEGG pathway enrichment of the 311 common targets of GS-Rd and AD were investigated. As shown in Figure 4B, the anti-AD target genes of GS-Rd are enriched in six major signal pathways, including human diseases, organismal systems and genetic information processing. The top 20 pathways (*p* < 0.05; Figure 4C) showed that GS-Rd intervention on AD targets involves multiple signaling pathways, including MAPK (mitogen-activated protein kinase) signaling pathway, Ras signaling pathway, PI3K-Akt signaling pathway and FOXO signaling pathway. Results suggest that GS-Rd has a wide range of pharmacological effects in AD, including regulating pathways in cancer, insulin resistance and tumor-immune environments.

### 3.3 Analysis of molecular docking results of GS-Rd and AD-related genes

**FIGURE 5:**
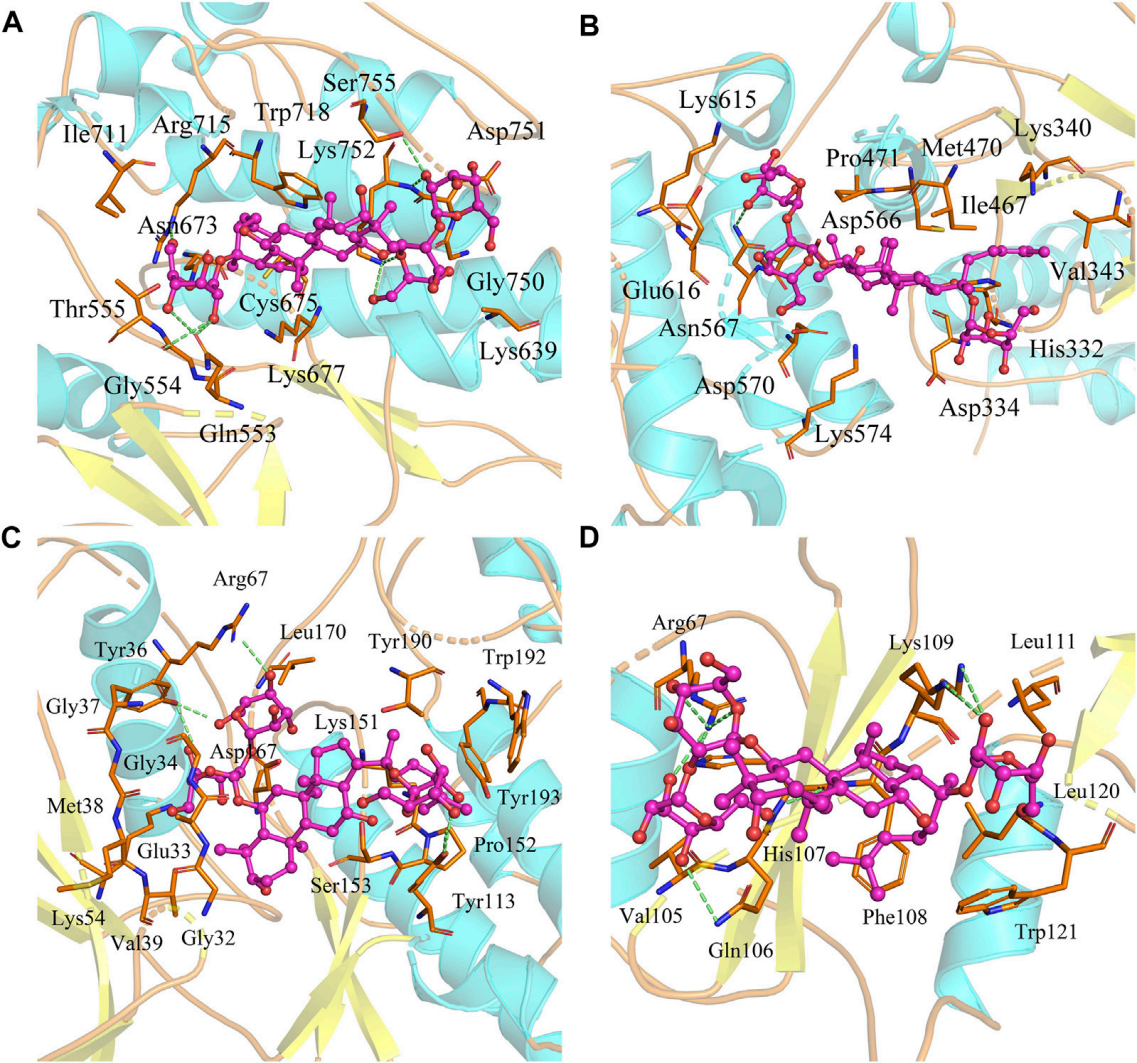
Molecular docking conformation of GS-Rd and hub genes. **(A)** JAK2 (docking affinity score: 7.5) **(B)** STAT3 (docking affinity score: 6.7) **(C)** MAPK1 (docking affinity score: 8.1) **(D)** GRB2 (docking affinity score: 6.4).

Based on the biological annotation results of the core targets, we suspected that the GS-Rd gene interferes with AD-related genes and the JAK-STAT signaling pathway and MAPK signaling pathway. In this study, GS-Rd was analysed together with hub genes (JAK2, STAT3, GRB2 and MAPK1) using molecular docking. The molecular docking affinities of the four core genes are all less than -5 kJ/mol (Figure 5); the outcomes of molecular docking demonstrated that GS-Rd may interact closely with related targets, confirming the component-target prediction findings. GS-Rd may interact with JAK2, STAT3, GRB2 and MAPK1 directly, thereby helping to regulate the biological function regulation process of AD.

### 3.4 GS-Rd extends lifespan and increases heat stress resistance in *C.* elegans with muscle-specific expression of Aβ

**FIGURE 6:**
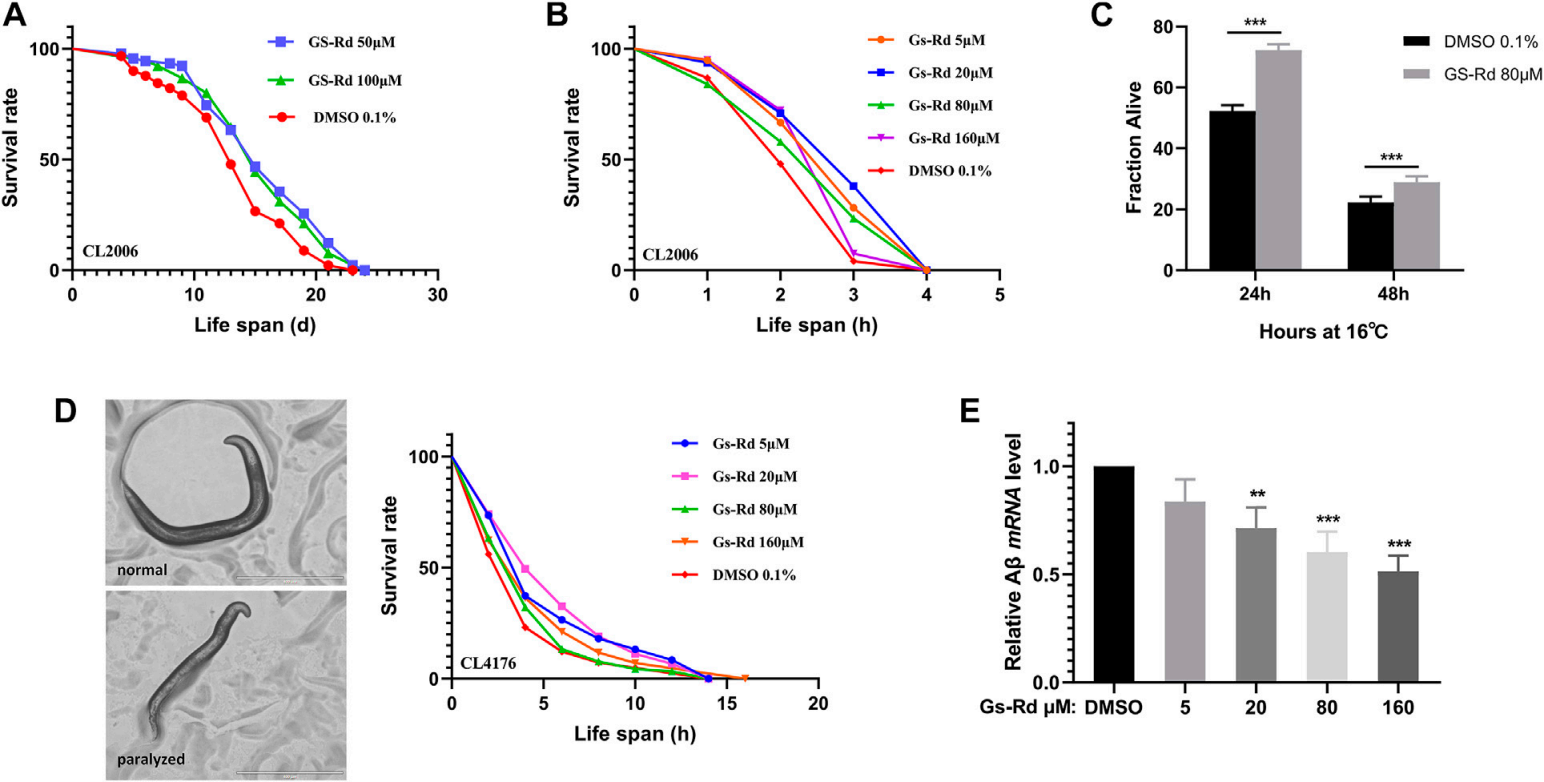
Effects of GS-Rd on the lifespan and heat tolerance of CL 2006. **(A)** Lifespan of GS-Rd treated or no treat CL2006 worms. **(B)** Survival rate of CL2006 worms in heat resistance experiments. Heat resistance experiments were carried out at 35°C and calculated by 1 h. **(C)** Survival rate of CL2006 worms in heat resistance recovery experiment. The experiments were carried out at 35°C for 7 h, then transferred to 16 °C and the number of dead worms was measured after 24 and 48 h. **(D)** Microscope images show CL4176 worms were paralyzed in larval status. **(E)** The Aβ mRNA level was measured by RT-qPCR. Error bars indicate SE. *p < 0.05, ***p < 0.01.

To strengthen the evidence for the particular roles that RD plays in the therapy of AD, the *C. elegans* strains CL2006 and CL4176 were used as AD models. First, to ascertain whether GS-Rd shields *C. elegans* from the toxicity caused by Aβ, lifespan assay data was obtained and (Figure 6A) showed a significant rightward shift, suggesting that GS-Rd extended the lifespan of CL2006 worms. Then, heat resistance trials and heat resistance recovery experiments both showed that GS-Rd reduced the lethality of heat stress (Figures 6B, C). These findings indicated that GS-Rd slows aging and delays age-related degeneration in *C. elegans* with muscle-specific expressions of Aβ.

### 3.5 Aβ-induced paralysis is alleviated by GS-Rd in transgenic *C. elegans*

The CL4176 worms, which express Aβ in muscle cells, can be paralyzed in a temperature-sensitive manner. Our data demonstrated that GS-Rd could prevent CL4176 worms from paralyzing (Figure 6D left panel), the worms were unable to move except for the oropharynx. At the fourth hour, there were fewer paralyzed CL4176 worms in the GS-Rd group, and the time needed for all worms to become paralyzed was considerably longer in the GS-Rd group than in the control group (Figure 6D right panel). These results revealed that GS-Rd could delay the paralysis of CL4176 worms caused by Aβ deposition.

Next, to learn more about whether the delayed paralysis of CL4176 worms was related to the Aβ expression level, we measured the Aβ mRNA levels using RT-qPCR. The expression of Aβ mRNA was found to be significantly down-regulated after being exposed to various concentrations of GS-Rd (Figure 6E), indicating that GS-Rd was able to lessen the toxicity caused by Aβ. These findings indicate that RD’s ability to delay worm paralysis is achieved *via* decreasing Aβ mRNA level.

### 3.6 AD-associated pathways were enriched in RNA-Seq of GS-Rd treated CL4176

**FIGURE 7:**
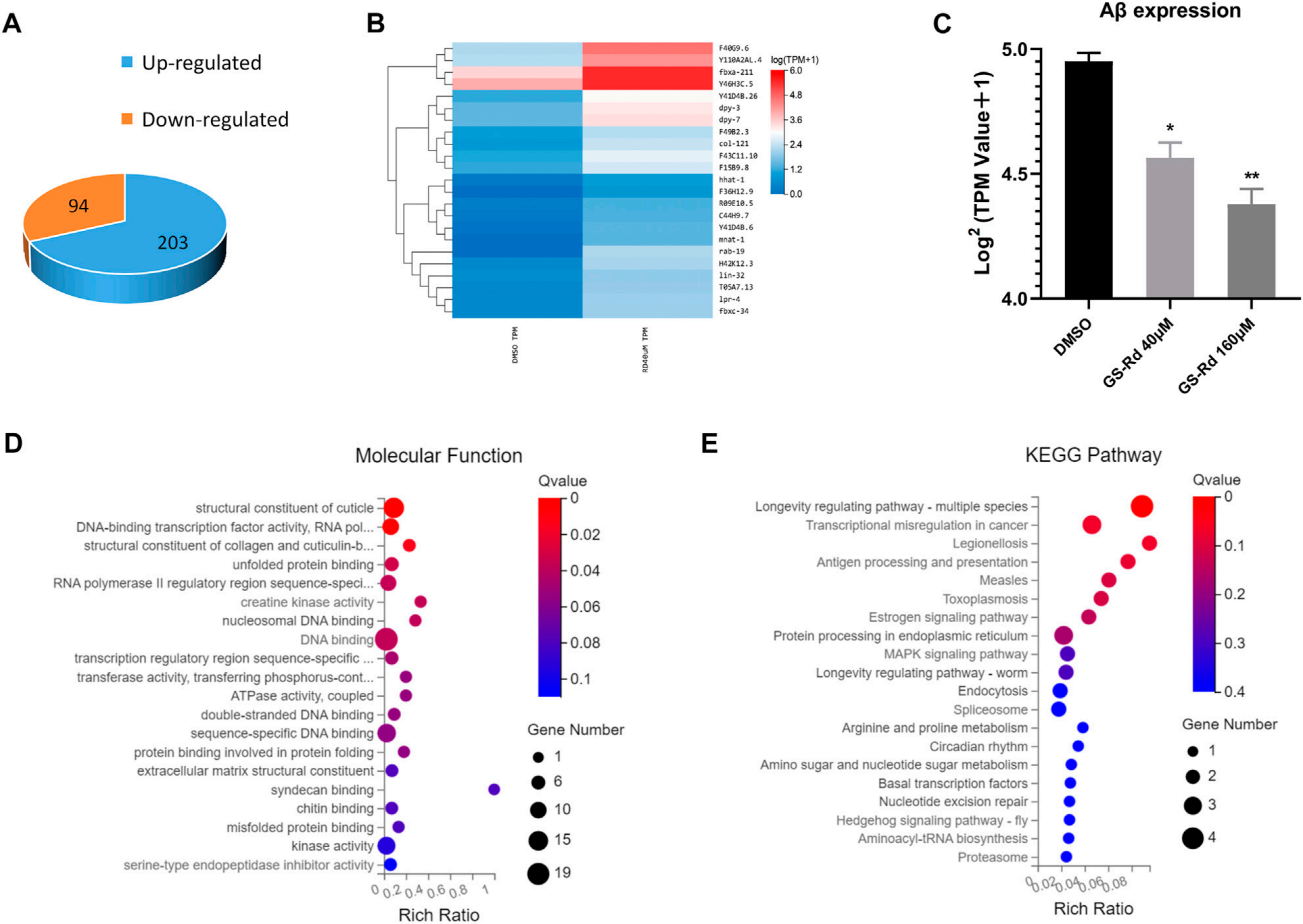
Analysis to the genes differentially expressed in GS-Rd treated CL4176 worms and the AD-associated pathways were enriched by RNA-Seq. **(A)**The number of differentially expressed genes between GS-Rd and DMSO treated CL4176 worms. log^2^ (GS-Rd/DMSO)>|1|; *p* < 0.05 was applied. **(B)** The heatmap of differentially expressed genes between GS-Rd and DMSO showed two main clusters of upregulated and downregulated genes. Each column represents an experimental condition and each row represent log^2^ Ratio value or log10 (FPKM+0.01) of a gene. **(C)**The Aβ mRNA expression level in GS-Rd and DMSO groups. **(D)** The GO analysis clarifies the molecular function of the differentially expressed genes in the CL4176 strain. **(E)** Top KEGG pathways enriched by the differentially expressed genes in GS-Rd treated CL4176.

Using RNA-Seq, it was possible to pinpoint the likely causes of the GS-Rd-treated CL4176 worms’ lower paralysis rate by identifying the altered gene expression and signaling networks. As shown in Figure 7A, the RNA-Seq results showed that 94 genes were significantly upregulated and 203 genes were significantly downregulated in 40 μM GS-Rd group [log^2^ (GS-Rd/DMSO)>|1|, *p* < 0.05]; the heatmap of differential gene clusters is shown in Figure 7B. The Aβ mRNA level decreased in 40μM and 160 μM GS-Rd treated CL4176 worms (Figure 7C).

We used GO enrichment analysis (https://metacpan.org/pod/GO::TermFinder) to identify the gene networks that were regulated in GS-Rd-treated CL4176. The aggregate of highly differentially expressed genes was clustered into the functions of DNA binding and kinase activity in molecular functions, transcription regulation process in biological process, cytoplasm and the nucleus in cell components (Figure 7D, Supplementary Data). The KEGG pathway enrichment analysis also showed that the MAPK signaling pathway (Figure 7E), which is well-known to be implicated in AD, was significantly impacted, as well as that protein processing in the endoplasmic reticulum and transcription regulation in cancer play roles in AD, this strongly suggests that GS-Rd reduces the synthesis of Aβ through the MAPK pathway, thereby reducing Aβ deposition.

### 3.7 GS-Rd increases oxidative stress resistance in transgenic *C. elegans*

**FIGURE 8:**
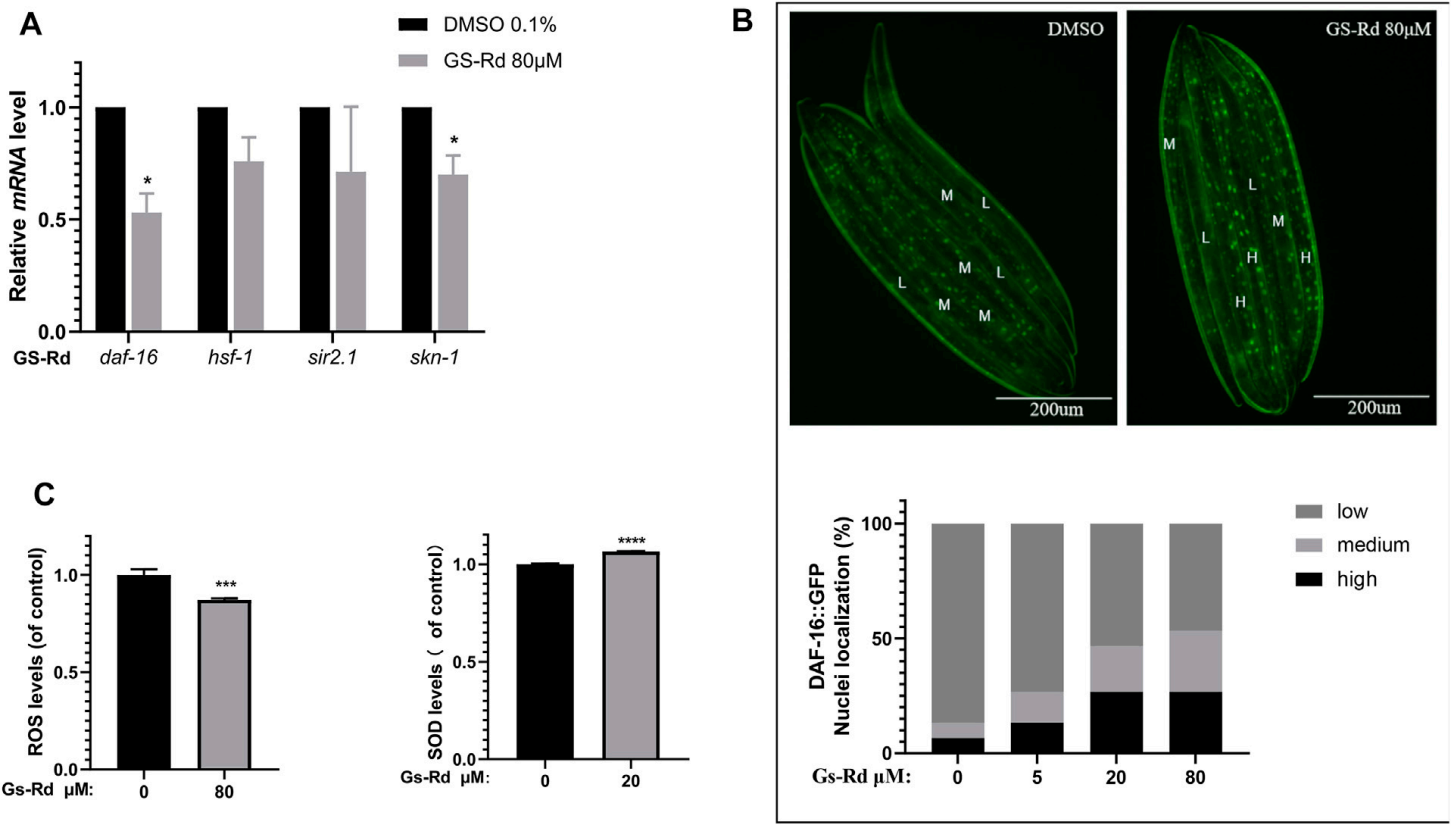
Effect of GS-Rd on oxidative stress related genes and ROS and SOD levels in **(C)** *elegans*. **(A)** RT-qPCR analysis of oxidative stress-related gene mRNA expression changes in GS-Rd-treated CL4176. **(B)** GS-Rd enhanced nuclear protein Daf-16 incorporation **(C)** 80 μM of GS-Rd decreased the ROS levels and increased the activity of SOD in CL4176 worms.

According to some research, the MAPK signal pathway can produce oxidative stress, which results in the deposition of Aβ and tau hyperphosphorylation, and can be caused by the production of neurotoxic inflammatory cytokines and reactive oxygen species (ROS) (Giraldo et al., 2014). To ascertain whether GS-Rd impacts specific oxidative stress-related signaling pathways linked to worm paralysis, some resistance-associated transcription factors were discovered using RT-qPCR. In GS-Rd-treated CL4176 worms, Figure 8A shows a significant decrease in *daf-16* and *skn-1*, which is in line with RNA-seq results (Supplementary Data). Therefore, we hypothesize that GS-Rd may be able to resist the harmful effects of Aβ in some way by downregulating *daf-16* and *skn-1* after administration. We then investigated whether GS-Rd alters the intracellular distribution of *daf-16*, as daf-16 can only enter the nucleus to trigger transcription of its target genes there. According to fluorescent microscopy images (Figure 8B upper panel), GS-Rd was able to exert *daf-16* transcript from the cytoplasm to the nucleus. We can see that GS-Rd significantly increased *daf-16* contents in the nuclei of TJ356 nematodes by using Image Pro Plus to analyze the relative fluorescence intensity of the worms (Figure 8B lower panel).

Since Aβ aggregation causes oxidative stress (Younus, 2018; Yang and Lian, 2020), increasing SOD and scavenging ROS can help to diminish the symptoms of Alzheimer’s disease. As shown in Figure 8C (left), 80 μM GS-Rd treatment significantly reduced the ROS level in CL4176 worms. It was revealed that decreasing the quantity of ROS in nematodes is what caused GS-Rd to delay the paralysis of CL4176 worms. Figure 8C (right) demonstrates that 20 μM of GS-Rd boosted SOD activity in CL4176 worms, suggesting that the ability of GS-Rd to postpone CL4176 worm paralysis may be connected to the enhancement of SOD activity.

## 4 Discussion

This study was conducted to explore the potential mechanism of ginsenoside Rd for the treatment of Alzheimer’s disease with the help of network pharmacology. We found that GS-Rd might perform an anti-AD effect by down-regulating the expression level of Aβ mRNA level based on the CL4176 worm experiment. 311 AD-related proteins were identified as prospective GS-Rd targets from the relevant database and the top 10 core targets were identified using topological analysis and linked to 136 signaling pathways, many of which were connected to the development of cancer, for example, the JAK-STAT signaling pathway, the MAPK signal pathway and other pathways associated with cancer. 311 potential targets exert therapeutic effects on AD through 29 biological processes, 11 molecular functions and 15 cellular compositions, including immune system processes, cell proliferation, transcriptional regulation, antioxidant activity, cells and membranes. It is suggested that GS-Rd is a multi-pathway and multi-target treatment for AD.

Alzheimer’s disease is the leading cause of dementia and is rapidly becoming one of the world’s most costly and fatal diseases. (Rudajev and Novotny, 2022). Extracellular senile plaques and intracellular tau protein tangles resulting from the accumulation of β-amyloid (Aβ) are key histopathological hallmarks of Alzheimer’s disease (AD), and the neurotoxicity of Aβ may play a central role in the pathogenesis of AD (Viola and Klein, 2015). Therefore, the key to treating Alzheimer’s disease is to limit Aβ neurotoxicity. Since it has good pharmacological activity, GS-Rd has attracted a lot of attention. Yan X (Yan et al., 2017) discovered that GS-Rd improved learning and memory in OVX rats by down-regulating the expression of β-secretase and Aβ *via* stimulating estrogen-like activity. However, there are few reports of GS-Rd preventing Alzheimer’s disease by inhibiting Aβ deposition, and the molecular mechanism of treatment is still poorly understood, therefore, we empirically validated the hypothesis that was generated by network pharmacology regarding the potential mechanism of GS-Rd treatment of AD, providing a foundation for further studies. Using the CL4176 transgenic nematode model, we confirmed the results predicted by network pharmacology using the CL4176 transgenic AD model. The frequency of paralyzed worms in the GS-Rd-treated group was considerably lower than that in the control group, demonstrating that GS-Rd might delay the paralysis of CL4176 worms caused by Aβ deposition. The expression of Aβ mRNA was significantly down-regulated in worms that were treated with different concentrations of GS-Rd, indicating that GS-Rd was able to reduce Aβ-induced toxicity. In the meantime, we performed RNA-Seq assays on GS-Rd-treated CL4176 nematodes and showed that GS-Rd was able to regulate the expression of a variety of genes, including 94 up-regulated genes and 203 down-regulated genes. GO analysis of the genes with more than a 2-fold difference in expression revealed that these genes were mainly involved in the regulation of DNA transcription and enzymatic activity, and KEGG analysis showed that these significantly altered genes were concentrated in the MAPK signal pathway, which is closely related to AD and various cancer-related signal pathways. These results were highly consistent with the results predicted by network pharmacology.

Mitogen-activated protein kinase (MAPK) is a group of highly conserved serine/threonine proteases in eukaryotes that regulate cell proliferation, differentiation, growth and apoptosis (Heneka et al., 2010). Previous research has identified a role for MAPK in the pathogenesis of AD, inducing Aβ aggregation, oxidative stress, neuroinflammation, mitochondrial dysfunction and memory impairment. For example, activated JNK and P38 are highly expressed in the brains of AD patients (Area-Gomez et al., 2018), JNK also co-localizes with Aβ (Song et al., 2018), and the activation of JNK in APP/PS1 double transgenic mice resulted in a significant increase in senile plaques and neurogenic fibrillary tangles, as well as neuronal apoptosis (Fang et al., 2018). These studies suggest that the activation of MAPK promotes the development of AD and, therefore, the use of inhibitors of MAPK may be an effective way to treat AD. Animal and cellular studies have shown that inhibitors of MAPK do reduce Aβ deposition (Hardy and Allsop, 1991), tau hyperphosphorylation (Hardy and Higgins, 1992) and neuronal apoptosis (Karran and De Strooper, 2016). However, MAPK inhibitors are extremely toxic and do not cross the blood-brain barrier; it is not practical to use these MAPK inhibitors to treat AD. In order to find signaling molecules that are closely related to AD pathogenesis and thus provide new targets and approaches for the prevention and treatment of AD, a deeper understanding of the MAPK signaling pathway and the hunt for signaling molecules that are directly associated with AD pathogenesis is necessary. Combined with RNA-seq data, we found that GS-Rd was able to inhibit the mRNA levels of Aβ and resistance genes *daf-16* and *skn-1* in CL4176 nematodes by suppressing the MAPK signaling pathway and reducing the ROS content and SOD-3 content in CL4176. According to data from fluorescence microscopy, GS-Rd encourages DAF-16 entrance into the nucleus to stimulate the expression of a number of genes.

Using network pharmacology analysis, we identified 311 protein targets common to both GS-Rd and AD and certain targets of GS-Rd (e.g., SRC, MAPK1, JAK2, STAT3, and GRB2), which are known to be important in AD biology (Li et al., 2019; Reichenbach et al., 2019; Wang et al., 2019; Yang et al., 2020; Roberts et al., 2021). Some highly connected nodes in the network were identified by a topological network algorithm. For example, STAT3 interacts with four other proteins (JAK2, EGFR, SRC and GRB2), each of which also exhibits high connectivity. STAT3 is a member of the STAT protein family that mediates multiple intracellular signaling pathways and is involved in many biological processes, including cell proliferation, survival, differentiation and angiogenesis (Hanlon et al., 2019). STAT3 protein is a key factor in the activation of the JAK-STAT pathway, and it has been found (Boza-Serrano et al., 2018) that JAK/STAT is directly involved in inflammatory responses induced by microglia. Further, Chintapaludi SR et al. (Chintapaludi et al., 2020) identified the JAK/STAT pathway as a possible regulator of plaque deposition. Several studies have linked the JAK/STAT pathway to amyloidosis and AD, and our molecular docking results showed that GS-Rd can interact directly with JAK2 and STAT3, suggesting that GS-Rd may exert anti-AD activity by regulating Aβ deposition through the modulation of the JAK/STAT pathway. Some studies have found that (Tóthová et al., 2021) the JAK-STAT signaling pathway can activate the downstream MAPK pathway and the PI3K-AKT pathway to further regulate intracellular protein synthesis, affect cell proliferation and cell cycles and other biological functions to regulate the cell growth process. Meanwhile, the results of molecular docking of GRB2 and MAPK1, the key proteins of the MAPK pathway and the PI3K-AKT pathway, showed that they could directly interact with GS-Rd, and the 311 core targets of KEGG were enriched to the MAPK pathway and the PI3K-AKT pathway, which suggested that we could next activate the downstream MAPK and PI3K-AKT pathways through the JAK-STAT signaling pathway.

In conclusion, this study found that GS-Rd regulates APP transcription through the MAPK pathway to reduce Aβ mRNA levels, thereby reducing the production of Aβ and ROS in AD and providing a new target for the treatment of AD. According to the results discussed above, GS-Rd can operate as a neuroprotective agent by reducing the neurotoxic effects of Aβ through anti-oxidative stress. This suggests that GS-Rd may be a novel target for the treatment of AD. It should be acknowledged that further studies are needed to determine how the MAPK signaling pathway has been altered.

## Data Availability

The data presented in the study are deposited in the NCBI repository (http://www.ncbi.nlm.nih.gov/bioproject/907863), accession number PRJNA907863.
