# Supplementary material for: Ginsenoside Rd protects transgenic Caenorhabditis elegans from β-amyloid toxicity by activating oxidative resistant
Source: Front Pharmacol. 2022 Dec 15;13:1074397. doi: 10.3389/fphar.2022.1074397 (PMC9797510; doi:10.3389/fphar.2022.1074397)

Supplementary Data

Supplementary Table 1. Sequence of primers for RT-PCR.

Gene	Sequence(5'→3')
act-1	forward:TCGGTATGGGACAGAAGGAC-
	reverse: CATCCCAGTTGGTGACGATA
Aβ	forward:CCGACATGACTCAGGATATGAAGT
	reverse:ACCATGAGTCCAATGATTGCA
Daf-16	forward: TTTCCGTCCCCGAACTCAA
	reverse: ATTCGCCAACCCATGATGG
SKN-1	forward: AGTGTCGGCGTTCCAGATTTC
	reverse: GTCGACGAATCTTGCGAATCA
hsf-1	forward: TTGACGACGACAAGCTTCCAGT
	reverse: AAAGCTTGCACCAGAATCATCCC
sir2.1	forward: AGAACGCGCATTTCGCCATATTAAG
	reverse: ATACTGACACTCCAGCGCCAG

Supplementary Figure 1.1 The GO analysis clarifies the biological process of the differentially expressed genes in the CL4176 strain.

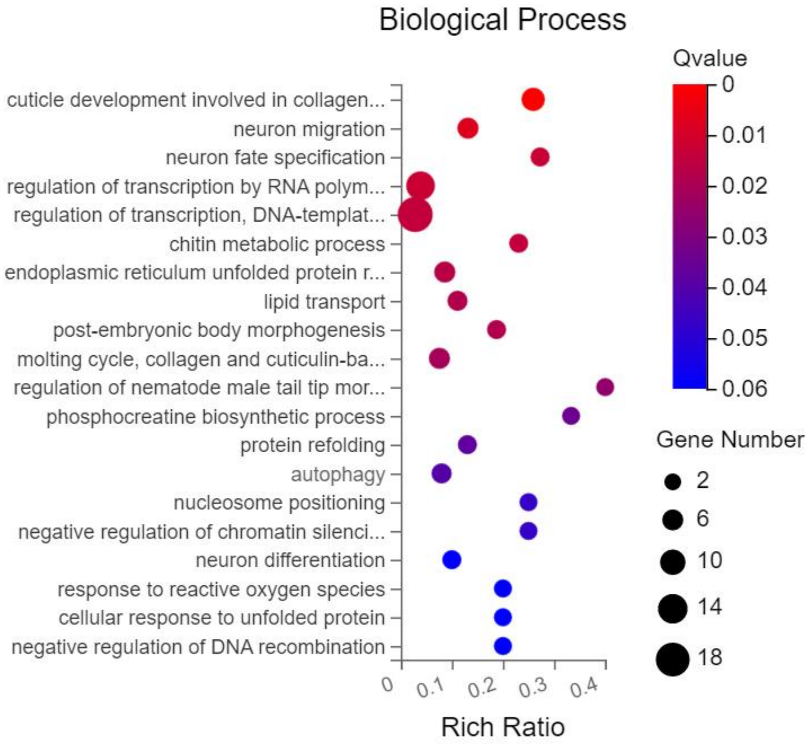

Supplementary Figure 1.2 The GO analysis clarifies the cellular component of the differentially expressed genes in the CL4176 strain.

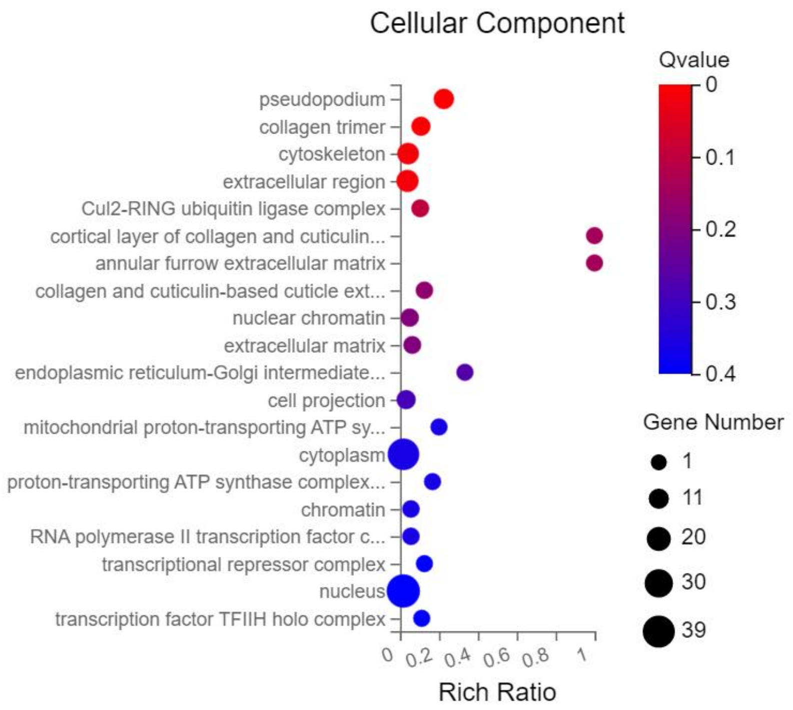

Supplementary Figure 3. RNA-seq analysis of daf-16, sir2.1, hsf-1, skn-1 mRNA expression levels in GS-Rd group and DMSO group.

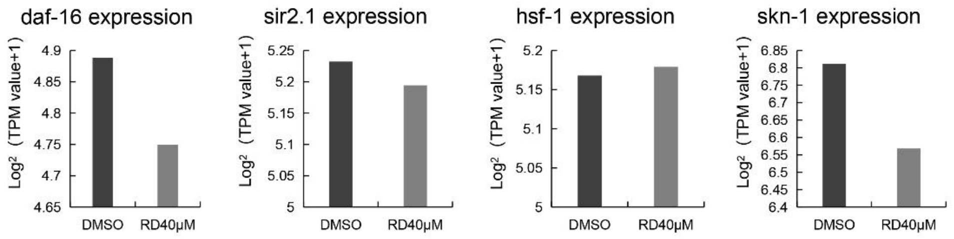

Supplement: Supplementary file 2 [file DataSheet1.PDF]
